# Evaluating Physical and Perceptual Responses to Exergames in Chinese Children

**DOI:** 10.3390/ijerph120404018

**Published:** 2015-04-13

**Authors:** Patrick W. C. Lau, Yan Liang, Erica Y. Lau, Choung-Rak Choi, Chang-Gyun Kim, Myung-Soo Shin

**Affiliations:** 1 Department of Physical Education, Hong Kong Baptist University, Kowloon Tong, Hong Kong, China; E-Mail: wclau@hkbu.edu.hk; 2 Department of Exercise Science, Arnold School of Public Health, University of South Carolina, Columbia, SC 29208, USA; E-Mail: lauy@email.sc.edu; 3 Sports Management, Division of Sport Science, College of Science and Technology, Konkuk University, ASI KR KS001, Chungju, Korea; E-Mail: cmas@nate.com; 4 Division of Sports Science, College of Arts and Design, Gachon University, ASI KR KS009, Seongnam, Korea; E-Mail: ckkim@gachon.ac.kr

**Keywords:** exergame, energy expenditure, heart rate, physical exertion, overweight, children

## Abstract

*Purpose*: The primary objective of this study was to examine whether exergames could help children reach the recommendations for PA and cardiorespiratory fitness regarding exercise intensity. Differences in perceived physical exertion, EE, VO_2_, and HR between normal weight (NW) and overweight (OW) children participating in exergames were also examined. *Methods*: Twenty-one children (age: 10.45 ± 0.88) were assessed for EE, VO_2_ and HR during rest, in a maximal treadmill test, and while playing different exergames. Ratings of perceived exertion (RPE) (category range: 0 to 10) were also measured during exergaming. Three types of exergames were examined: running, table tennis, and dancing. These games were either performed on a Chinese game console, I-Dong, or another well-developed Western game console (Sony PlayStation 3 or Nintendo Wii). *Results*: Exergaming resulted in EE (kcal/min) from 2.05–5.14, VO_2_ (mL/kg/min) from 9.98–25.54, and HR (beats per minute) from 98.05–149.66. Children reported RPE ranging from 1.29 to 5.29. The Chinese exergame, I-Dong Running, was the only game in which children reached a moderate intensity and met the recommended minimum VO_2_reserve (50%) for cardiorespiratory fitness. *Conclusion*: Exergames could provide alternative opportunities to enhance children’s physical activity. They could be used as light-to-moderate PA, and with exergames, children can even reach the recommended intensity for developing and maintaining cardiorespiratory fitness.

## 1. Introduction

Childhood obesity has serious negative consequences for health and wellbeing both during childhood and in later adult life [1]. The rise in childhood excess body weight and obesity, which has been declared a global public health burden [2], is associated with decreasing physical activity (PA) and increasing sedentary behavior [3]. In addition, the other benefits of regular PA for physical and mental health have been well documented [4,5,6]. A dose-response relationship has been observed, with more PA associated with greater health benefits [7]. International guidelines have recommended that children should engage in at least 60 min of moderate-to-vigorous physical activity (MVPA) per day [6]. However, many children do not achieve the PA recommendations [8,9,10]. Physically inactive children were associated with negative health outcomes such as a lower level of cardiovascular fitness [11], higher body fat, higher risk of cardiovascular and metabolic diseases, worse bone health and worse mental health [6,7,12]. Existing guidelines recommend that children should minimize the time spent being sedentary, and particularly limit use of screen-based sedentary behaviors (e.g., television viewing, video game playing, and computer use) to no more than two hours per day [13,14]. However, children are not likely to give up this type of sedentary habit to improve health [15], and they spend approximately 3–5 h a day in these screen-based activities [16]. Therefore, attempts have been made to increase children’s PA by transforming sedentary screen time into active screen time.

Exergames, which integrate exercise and gaming entertainment [17], might provide an attractive option for displacing sedentary screen time and increasing PA in children. They have been used in previous controlled trials in children for one or more of the below purposes: to increase PA and/or replace sedentary behaviors [18,19,20,21,22,23,24] and to prevent or treat childhood obesity [25,26,27,28]. Previous studies produced inconclusive results due to the different targeted health outcomes and the heterogeneity/intensity of the exergames in children’s PA and cardiovascular responses studies [29,30]. Unnithan and his colleagues [31] conducted a study on the energy cost of playing a dance simulation video game, and they found that the dance simulation game could not reach the recommended intensity for developing and maintaining cardiorespiratory fitness suggested by American College of Sports Medicine (ACSM). Consequently, it is valuable to examine whether other exergames could reach the recommended intensity.

Other than the physical responses, the perceptual responses to exergames might also be important for the potential use of exergames in improving children’s health. Perceived exertion was defined as either an individual’s subjective rating of the work intensity when exercising or how the individual interpreted their feeling during the exercise workload [32]. This concept might help explore the possible impact and difference between “what you are doing” and “what you think you are doing” in exercise. Children usually indicated that they enjoyed exergaming [33,34], which might result in an underestimated perceived exertion and sustained engagement compared to other activities of the same intensity. In the study of Bartlett *et al.* [35] and Sim *et al.* [36], a higher rating of perceived exertion (RPE) was accompanied by higher perceived enjoyment in adults after an intensive intermittent running exercise. Stork *et al.* [37] also found that listening to music could enhance and maintain intensive exercise in young adults. These findings might apply to children, and exergames could have a similar effect to music on children’s RPE and enjoyment. However, RPE compared to actual exertion level has seldom been examined in children in previous studies. It is imperative to determine the physical and psychological responses to exergames before using them in childhood obesity intervention. Furthermore, the majority of previous research trials have been conducted in Western countries. Very few research studies on this topic have been conducted with Asian or Chinese children.

Therefore, the present study intended to examine the EE, oxygen uptake (VO_2_), HR, and RPE through three different types of exergames that have been popular in Hong Kong primary schools and simulate three forms of sport activities, including running, table tennis, and dancing. In addition, metabolic equivalent values (METs) and VO_2_ reserve during exergaming were derived and compared with children’s PA recommendations from the World Health Organization and the ACSM. The primary objective of this study was to examine whether exergames could help children to reach the recommendations for PA and cardiorespiratory fitness regarding exercise intensity. Differences in perceived physical exertion among participants and the children’s EE, VO_2_, HR with exergames between normal weight (NW) and overweight (OW) children were also examined. The present study might be able to contribute to the understanding of the impact of exergames on Chinese children. To our knowledge, none of the chosen games have been tested in Chinese children.

## 2. Methods

### 2.1. Participants

Children who had no physical or intellectual issues that might prevent them from being active were recruited from one primary school in Hong Kong via school teachers. Telephone contacts were conducted with parents to confirm interest before they were invited to visit a laboratory at Hong Kong Baptist University. Both participants and parents provided written consent at the first visit. There were no physical or intellectual issues that might prevent participants from undertaking this study. This study was approved by the Senate Committee on the Use of Human and Animal Subjects in Teaching and Research, Hong Kong Baptist University.

### 2.2. Procedures

After consent forms were signed by children and their parents, a pre-test questionnaire was employed to screen the medical history of participants and to record any medications using answers from children and their parents. Children with physical disability or chronic diseases were excluded from the study. Study procedures were then explained to the participants. Standing heights were measured barefoot to the nearest 0.1 cm using a wall-mounted metal anthropometer (Novel Products, Rockton, USA). Weights were assessed to the nearest 0.1 kg using an electronic scale (Tanita, Model WB-100, Tokyo, Japan). Maximum oxygen uptake (VO_2max_) was assessed after the anthropometric measurements. VO_2max_ was determined using a treadmill ramp test protocol. Children started walking at 3.2 km/h, and the speed gradually increased to a speed allowing children to run during a two-minute warm-up session. Following the warm-up, the test began, and the gradient was increased to 4% after four minutes. Every two minutes thereafter, the gradient was increased by 2% until volitional exhaustion. Every two minutes after the test began, children were asked to point to the RPE value on a children's OMNI scale of perceived exertion [38], which has a developmentally numerical category format ranging from 0 (not tired at all) to 10 (very, very tired) with both pictorial and verbal descriptors. Criteria for maximal performance were as follows: (1) maximal heart rate of 200 beats per minute (bpm) or (2) a respiratory exchange ratio of 1.0 [39]; these values were attained at least once during the gradient increase.

After the treadmill test, the children were monitored for at least 10 min and then provided refreshment. Children played the selected exergames: I-Dong running (ID RUN), Nintendo Wii Obstacle Course (WII RUN), I-Dong Table Tennis (ID TT), Sony PlayStation 3 Table Tennis (PS TT), I-Dong Dancing (ID DAN), and Nintendo Wii Rhythm Kung-Fu (WII DAN), for a familiarization period lasting approximately 30 min. Among these games, running games mainly required lower-body movement; children needed to jump to avoid moving bars in ID RUN and overcome obstacles in WII RUN. An elastic belt with an optical identification point was fixed on the right knee of each participant for capturing movement in ID RUN. The Wii Balance Board™ was used to capture the movement of children during WII RUN playing. Children played ID TT using a real racquet with an attached optical identification point. PlayStation^®^Move Motion Controller (a hand held game controller) was used to simulate a racquet to play PS TT. Children held controllers in both hands and moved their arms following the instructions on screen while playing the two dancing games. Lower body movement was required and captured by the Wii Balance Board^TM^ in WII DAN but not in ID DAN.

I-Dong^TM^ (Taishan Online, Shen Zhen, China), which means “Love to be active” in Chinese, exergames have been available since 2007. I-Dong exergames have several unique features compared to other exergames, including (1) the ability to be installed in personal computers as online games and (2) the use of optical identification points, which can be attached conveniently on the human body and peripheral equipment (such as real racquets instead of game remote controllers) for interaction between body movements and exergames.

After obtaining the VO_2max_ and undergoing a familiarization session, an additional game playing session was undertaken at least five days later. During the game playing sessions, children came to the lab to play the six exergames in a previously set random order to avoid introducing systematic bias in the estimation of the measures. Participants came to the lab two times to complete the game playing sessions, which meant that they played three games for each visit. Children were asked to fast except for water at least four hours before coming to the lab. At the beginning of each game session, children were requested to lie down in the supine position for twenty minutes to assess their resting energy expenditure (REE). After having a refreshment, the children played each exergame for ten minutes following the same protocol. Running games started at the easiest level and processed through more advanced levels within 10 min. The same three songs were chosen for ID DAN. There were no different levels in table tennis games and WII DAN. Children were asked to restart the same exergame when it ended before the end of 10-minute measuring period. After playing one game, children were asked to sit down and rest for approximately five minutes. Meanwhile, the RPEs of exergaming were evaluated. Each participant’s heart rates was monitored during rest and needed to decrease to 90 bpm or less before starting the next game.

For all assessments, VO_2_ was assessed continuously using a portable indirect calorimetry respiratory gas analysis system MetaMax3B (Cortex, Biophysik, Leipzig, Germany) breath-by-breath. Prior to each test, calibration was conducted according to the standard guidelines. Standard gases of known concentrations (15% O_2_ and 5% CO_2_) were used to calibrate the oxygen and carbon dioxide sensors. A 3-L syringe was used to calibrate the flow sensor. A proper-sized face mask (Hans Rudolf, Kansas City, KS, USA) for children was used and attached to a turbine flow sensor to measure the volume of the respiratory air. Heart rate was continuously measured by a Polar^®^ heart rate monitor (Lake Success, NY, USA) during assessments.

### 2.3. Data Analyses

BMI was computed (weight in kilograms divided by height in meters squared). Overweight status was determined by the age and sex-specific BMI cut-off points [40].

The last minute data of the treadmill test were derived to estimate VO_2max_. The last two minutes data of resting were derived for analyses. The last five minutes data of each game playing were derived for estimating EE and heart rate during exergaming.

Energy expenditure was converted from VO_2_ using the established constant (1 L O_2_ = 4.9 kcal) [41]. Child-specific METs were calculated by dividing the respective activity VO_2_ by resting VO_2_ [42]. METs were used to interpret intensities of activities: sedentary (≤1.5 METs) [43], light (1.6–2.9 METs) [43], moderate (3–6 METs) [44], and vigorous (>6 METs). VO_2reserve_ and METs were computed for describing intensities of the tested exergames. VO_2_ reserve was calculated using the respective activity VO_2_-resting VO_2_/VO_2max_-resting VO_2_ [31]. The mean values of each measure across games were compared using repeated-measures analyses of variance with Bonferroni *post hoc* comparisons. A *p*-value was set at 0.05. Statistical analyses were conducted using SPSS version 21.0 (IBM Inc. Armonk, NY, USA).

## 3. Results

Twenty-one children (17 males and four females), ages 9–12 years (10.45 ± 0.88) participated and completed all research sessions. Among them, 13 children were normal weight and eight were overweight (Table 1). The overweight group had a significantly (*p* < 0.05) higher weight and BMI.

**Table 1:** Participants’ characteristics by weight status.

Age & Weight Status	Total	OW (n = 8)	NW (n = 13)	*p*
Age (years)	10.45(0.88)	10.37(1.03)	10.50(0.82)	0.76
Weight (kg)	42.50(8.35)	48.95(9.18)	38.54(4.71)	0.02
Height (cm)	144.64(6.14)	144.49(7.14)	144.73(5.75)	0.93
BMI	20.20(2.98)	23.23(2.37)	18.34(1.29)	<0.001

Note: Values are the means (SD).

### 3.1. Physical and Perceptual Responses to Six Exergames

Exergaming with ID RUN consistently resulted in the highest values, including VO_2_, VO_2reserve_, absolute EE, adjusted EE, HR, METs, and RPE (Table 2). In contrast, exergaming with ID DAN consistently resulted in the lowest values in all outcomes.

There were no significant differences (*p* > 0.05) in EE, VO_2_, heart rate, Vo_2reserve_, or METs among the ID DAN, WII DAN, ID TT, and PS TT exergames. WII RUN and ID RUN resulted in significantly (*p* < 0.01) higher values in all physical responses than the previously mentioned four games.

**Table 2:** Physical and perceptual responses for each condition.

Condition	VO_2_ (mL/kg/min)	HR (bpm)	RPE	Absolute EE (kcal/min)	Adjusted EE (kcal/kg/min)	METs	VO_2_reserve (%)
Rest	6.85 (1.06)	79.20 (9.31)					
VO_2_max test	41.62 (5.72)	192.92 (8.69)	8.10 (1.51)				
ID RUN	25.54 (7.96) ^b^	149.66 (20.68) ^b^	5.29 (1.98) ^b^	5.14 (1.39) ^b^	0.13 (0.39) ^b^	3.71 (0.97) ^b^	53.98 (20.59) ^b^
WII RUN	17.74 (2.42) ^a^	122.83 (14.14) ^a^	2.57 (1.54)	3.65 (0.64) ^a^	0.09 (0.01) ^a^	2.64 (0.41) ^a^	31.86 (7.31) ^a^
ID TT	11.26 (2.72)	99.98 (9.44)	2.19 (1.69)	2.31 (0.56)	0.06 (0.13)	1.67 (0.34)	12.79 (5.94)
PS TT	11.88 (3.09)	102.33 (12.43)	2.10 (1.51)	2.45 (0.71)	0.06 (0.02)	1.76 (0.52)	13.94 (7.17)
ID DAN	9.98 (2.96)	98.05 (20.95)	1.29 (1.35)	2.05 (0.63)	0.05 (0.01)	1.53 (0.52)	9.35 (7.86)
WII DAN	11.23 (2.86) ^c^	101.58 (9.50)	2.43 (1.47)	2.30 (0.61) ^c^	0.06 (0.01)^c^	1.70 (0.43)	13.16 (7.65) ^c^

Note: Values are the means (SD). ^a^ Significantly (*p* < 0.01) higher than ID TT, PS TT, ID DAN, and WII DAN. ^b^ Significantly (*p* < 0.01) higher than ID TT, PS TT, ID DAN, WII DAN, and WII RUN. ^c^ Significantly higher than ID DAN (*p* < 0.01).

There were no significant differences (*p* = 1.00) in the RPE among the ID TT, PS TT, WII DAN, and WII RUN exergames. Children rated a higher exertion during playing WII RUN than ID DAN (*p* = 0.04). In addition, the RPE of WII DAN was marginally significantly higher than that of ID DAN (*p* = 0.05). The RPE of ID RUN was significantly higher than that of any other game (*p* < 0.01).

### 3.2. Comparison of Normal-Weight and Overweight Children in Physical and Perceptual Responses to Exergames

Overweight children spent significantly (*p* < 0.001) more energy during rest than the NW children, but the resting VO_2_ were similar when controlled for body mass (Table 3). The average VO_2max_ of NW children was significantly (*p* = 0.01) higher than that of OW children, whereas the energy spent during the last minute of the treadmill test was similar among the children.

There were no significant (*p* > 0.05) differences between NW and OW children in absolute EE and VO_2_ during exergaming (Table 3).

The average HRmax of OW children (n = 6) was 189.07, and the average HRmax of NW children (n = 10) was 195.22. There were no significant (*p* > 0.05) differences between NW and OW children in HR during any condition (Table 4).

**Table 3:** Energy expenditure and VO_2_ by weight status for each condition.

Activity	EE (kcal/min)			VO_2_ (mL/kg/min)		
	NW (n = 13)	OW (n = 8)	*p*	NW (n = 13)	OW (n = 8)	*p*
Rest	1.29 (0.10)	1.57 (0.08)	<0.001	6.90 (0.84)	6.76 (1.42)	0.78
VO_2max_ test	8.20 (1.16)	9.16 (1.98)	0.18	43.75 (6.11)	38.17 (2.72)	0.01
ID RUN	5.08 (1.15)	5.23 (1.81)	0.82	27.50 (7.71)	22.36 (7.78)	0.16
WII RUN	3.49 (0.60)	3.91 (0.64)	0.15	18.50 (2.24)	16.50 (2.31)	0.06
ID TT	2.19 (0.59)	2.50 (0.47)	0.22	11.62 (3.00)	10.67 (2.25)	0.45
PS TT	2.3 4(0.63)	2.63 (0.82)	0.38	12.53 (3.57)	10.82 (1.83)	0.16
ID DAN	1.95 (0.71)	2.22 (0.45)	0.35	10.29 (3.41)	9.48 (2.14)	0.56
WII DAN	2.20 (0.63)	2.47 (0.58)	0.34	11.64 (2.97)	10.54 (2.73)	0.41

Note: Values are the means (SD).

**Table 4:** Heart rate by weight status for each condition.

Activity	HR (bpm)		
	NW (n = 13)	OW (n = 8)	*p*
Rest	77.94 (10.25)	81.24 (7.73)	0.44
VO_2max_ test	195.22 (9.63)	189.07 (5.59)	0.18
ID RUN	152.86 (20.21)	144.46 (21.73)	0.38
WII RUN	122.10 (7.86)	124.03 (21.52)	0.81
ID TT	100.80 (11.17)	98.63 (6.12)	0.62
PS TT	103.61 (14.99)	100.25 (6.95)	0.56
ID DAN	94.99 (22.58)	103.03 (18.28)	0.41
WII DAN	101.71 (9.96)	101.38 (9.37)	0.94

Note: Values are the means (SD).

There were no significant (*p* > 0.05) differences between NW and OW children in RPE during any condition (Table 5).

**Table 5:** Ratings of perceived exertion by weight status for each condition.

Activity	RPE		
	NW (n = 13)	OW (n = 8)	*p*
VO_2max_ test	7.92 (1.32)	8.38 (1.85)	0.52
ID RUN	5.31 (1.60)	5.25 (2.61)	0.95
WII RUN	2.62 (1.45)	2.50 (1.77)	0.87
ID TT	2.23 (1.48)	2.13 (2.10)	0.89
PS TT	2.23 (1.59)	1.88 (1.46)	0.61
ID DAN	1.46 (1.27)	1.00 (1.51)	0.46
WII DAN	2.54 (1.51)	2.25 (1.49)	0.67

Note: Values are the means (SD).

There were no significant (*p* > 0.05) differences between NW and OW children in VO_2reserve_ and METs during any condition (Table 6).

**Table 6:** VO_2reserve_ and METs by weight status for each game.

Activity	VO_2reserve_ (%)			METs		
	NW (n = 13)	OW (n = 8)	*p*	NW (n = 13)	OW (n = 8)	*p*
ID RUN	55.53 (16.96)	51.46 (26.57)	0.67	3.89 (0.84)	3.40 (1.14)	0.27
WII RUN	32.11 (7.50)	31.45 (7.48)	0.85	2.72 (0.42)	2.50 (0.39)	0.25
ID TT	12.77 (6.57)	12.82 (5.16)	0.99	1.71 (0.39)	1.60 (0.22)	0.50
PS TT	14.91 (7.61)	12.36 (6.55)	0.44	1.84 (0.52)	1.65 (0.52)	0.42
ID DAN	8.99 (9.15)	9.93 (5.70)	0.80	1.55 (0.64)	1.49 (0.28)	0.80
WII DAN	13.25 (7.90)	13.01 (7.77)	0.95	1.75 (0.46)	1.63 (0.39)	0.55

Note: Values are the means (SD).

## 4. Discussion

To answer the primary research question of whether exergames could help children to reach the recommendations for PA and cardiorespiratory fitness regarding exercise intensity, the present study demonstrated that all exergames were light-to-moderate intensity activities based on the child-specific METs (1.53 to 3.71). This finding was consistent with the previous studies measuring EE of exergaming [29,30]. This intensity of exergames could be used as an alternative to help physically inactive children reach the recommended intensity for developing cardiorespiratory fitness and could possibly contribute to EE, MVPA or to displacing sedentary behavior. Moreover, ID RUN was the only exergame in the present study that reached moderate intensity (METs ≥ 3) and could be used as a tool to achieve the PA recommendations of children.

Regarding cardiorespiratory fitness, ID RUN was the only exergame that could reach the ACSM recommended minimum VO_2reserve_ (50%) for developing and maintaining cardiorespiratory fitness [31]. Because the chest strap of the heart rate monitor slipped down during the treadmill test, 24% (n = 5) of the data for maximum HR (HR_max_) was missing in the present study. Consequently, the percentage of HR_max_ during exergaming was not calculated and compared with the ACSM recommendations. However, VO_2reserve_ is a better index to describe the activity intensity than %HR_max_ [45]. According to one study [31], exergaming might reach the recommended %HR_max_ intensity (55%–65% HR_max_) for cardiorespiratory fitness, but it might not reach the recommended VO_2reserve_. Therefore, ID RUN could be considered to reach a more stringent standard of activity intensity to improve children’s cardiorespiratory fitness.

ID RUN warrants further studies to test whether it could be played by children sustainably to improve PA and cardiorespiratory fitness, especially in children who might have environmental barriers to engage in outdoor activities. In Hong Kong primary schools, the outdoor space (2 m^2^ per student) and outdoor playground area (2350 m^2^) are limited compared to primary schools in Korea (3000 m^2^), mainland China (5278 m^2^), and Japan (10,692 m^2^) [46]. The lack of outdoor space is because Hong Kong is one of the most densely populated cities in the world and is 100% urbanized [47,48]. The constraints of outdoor PA, such as facility accessibility, safety concerns, air pollution, and inclement weather, have worsened children’s PA development. Therefore, exergames that promote MVPA might provide a valuable opportunity for insufficiently inactive children in Hong Kong to become more physically active and to increase their cardiorespiratory fitness.

Although there were no significant differences between the dancing and table tennis games, EE and VO_2_ were significantly higher in WII DAN than ID DAN (*p* < 0.01) when lower-body movement was also required. This finding is consistent with previous studies showing that exergames requiring whole-body movement resulted in greater EE than those only requiring upper-body movement [49,50]. Given that running games resulted in the highest EE and heart rates across the six games, exergames that require more lower-body movement might result in increased EE.

RPE could be used as an index of exercise intensity too, but it is not as accurate as the physical indices [51]. Therefore, we did not use it as a criterion for the maximal performance of the treadmill test and did not interpret the RPE data for exergaming intensities. Although children exerted more energy playing WII RUN than table tennis and dancing exergames, children did not perceive this increased physical effort and or feel that the game was more difficult. This finding suggested that certain exergames might increase children’s EE but not necessary increased their ratings of perceived exertion. Based upon the literature of immersion study in exergames, it is speculated that once children immerse themselves into exergames, their perception of the game’s intensity might change and result in higher and longer motivation to play and exercise. This study suggested that there might be a “magic zone” within the light-to-moderate intensity activity that could produce higher intensity exercise but lower RPE compared to other exergames such as WII RUN, ID TT, PS TT (lower intensity, lower RPE) and the ID RUN (higher EE but also higher RPE). More research to explore this optimal zone is encouraged because it means that a more enjoyable experience might be produced with this specific intensity zone of exergames. Consequently, it could be a novel and significant zone for children to adopt/maintain their PA and enhance their health.

To answer the second research question, the results demonstrated that there were no significant differences between the NW and OW children in absolute EE, adjusted EE, HR and RPE across the exergames played. These findings were consistent with one study using indirect calorimetry to estimate EE during exergaming [52]. In contrast, two studies [31,53] found that OW children exerted more absolute EE than NW children when playing Dance Dance Revolution (DDR) (Konami Digital Entertainment, Redwood City, CA, USA). Therefore, research with future comparisons in absolute EE and adjusted EE, HR and RPE between different exergames should be sensitive to the intensity range between individual studies. Because none of the exergames employed in the present study have been previously examined, we presume that at least one of the reasons for the inconsistent findings in the literature could be the heterogeneity of exergames.

The present study had several limitations. First, the sample size was small. Although it was comparable to the previous laboratory studies measuring EE of exergaming, the small size might inhibit the comparison between NW and OW children due to insufficient statistical power. Second, more boys volunteered to take part in the present study. Although this might reflect the fact that children have different interests in exergames by gender, we were not able to examine gender differences in responses to exergames. Third, as in any laboratory study, we could not predict the exact EE when children play exergames freely, which might be different from the current results.

## 5. Conclusions

To conclude, exergames could provide alternative opportunities to enhance children’s physical activity. Exergames could be used as light-to-moderate PA and could help children reach the recommended intensity for developing and maintaining cardiorespiratory fitness. The running exergames might be promising for use in a field study to promote children’s PA and fitness. Finally, there is a significant activity intensity zone that might cause more EE with a lower RPE.
